# Acknowledgement to Reviewers of *Micromachines* in 2015

**DOI:** 10.3390/mi7010016

**Published:** 2016-01-21

**Authors:** Micromachines Editorial Office

**Affiliations:** MDPI AG, Klybeckstrasse 64, CH-4057 Basel, Switzerland; micromachines@mdpi.com

The editors of *Micromachines* would like to express their sincere gratitude to the following reviewers for assessing manuscripts in 2015.

We greatly appreciate the contribution of expert reviewers, which is crucial to the journal’s editorial decision-making process. Several steps have been taken in 2015 to thank and acknowledge reviewers. Good, timely reviews are rewarded with a discount off their next MDPI publication. By creating an account on the submission system, reviewers can access details of their past reviews, see the comments of other reviewers, and download a letter of acknowledgement for their records. In addition, MDPI has launched a collaboration with Publons, a website that seeks to publicly acknowledge reviewers on a per journal basis. This is all done, of course, within the constraints of reviewer confidentiality. Feedback from reviewers shows that most see their task as a voluntary and mostly unseen work in service to the scientific community. We are grateful to our reviewers for the contribution they make.

Abadie, J.
Abbyad, Paul
Abdelkefi, Abdessattar
Abdel-Rahman, Eihab
Abdul-Majid, Sawsan
Abell, Chris
Abramovich, Haim
Agah, Masoud
Agarwal, Gunjan
Agrawal, Nitin
Ahmad, Zeeshan
Aktakka, Ethem
Alan, Tuncay
Alba-Flores, Rocio
Alexander, Dennis R.
Alexeev, Alexander
Alfinito, E.
Ali, Syed Zeeshan
Aphale, Sumeet S.
Atkinson, Gary M.
Attinger, Daniel
Azam, Saeed Eftekhar
Baba, Yoshinobu
Bachmann, Herwig
Bahadur, Vaibhav
Baine, Paul
Banerjee, Ashis
Barniol, Nuria
Basu, Amar S.
Ben Azouzc, Aymen
Berdichevsky, Yevgeny
Bernini, R.
Bertassoni, Luiz Eduardo
Besharatian, Ali
Bombelli, Francesca B.
Bourouina, Tarik
Breadmore, Michael
Brousseau, Emmanuel
Brunye, Tad T.
Buividas, Ricardas
Burrows, Susan
Busta, Heinz
Buszewski, Bogusław
Caponetti, Laura
Cappelleri, Dave
Carugo, Dario
Casciati, Sara
Cattan, Eric
Ceyssens, Frederik
Chahal, Prem
Chahl, Javaan
Challamel, Noel
Chang, Honglong
Chang, Pei-Zen
Chao, Yimin
Chen, Chang-Cheng
Chen, Chia-Yuan
Chen, Chung-Nan
Chen, Hsiao-Chin
Chen, Jyh-Jian
Chen, Lingxin
Chen, Xi
Chen, Xianfeng
Chen, Yong
Chen, Yue
Cheng, Chao-Min
Cheng, Chiang-Ho
Cheng, Ya
Chia, Yen Lee
Chiang, Kai-Wei
Chiarot, Paul
Cho, Seong Yun
Cho, Young Hak
Choi, Chang
Chow, Jacky
chuang, Han-Sheng
Chung, Aram
Chung, Seungjun
Chung, Wen-Yaw
Cohen, Netta
Cong, Yongzheng
Corigliano, Alberto
D. García, Carlos
Da Silva, M. L. Alves
Dan Cho, Dong-Il
Delnavaz, Aidin
Delville, Jean-Pierre
Desmaris, Vincent
Di Bartolomeo, Antonio
Dickey, Michael
Didar, Tohid
Dietzel, Andreas
Dong, Lili
Drzazga, Anna
Edalati, Kaveh
Edwan, Ezzaldeen
Elshurafa, Amro M.
Endo, Tatsuro
Engelen, Peter-Jan
Falco, Gianluca
Fan, Xudong
Farajian, Amir A.
Fassi, Irene
Fatikow, Sergej
Feinberg, Adam W.
Felicetti, Luca
Femminella, Mauro
Feng, Zhi Hua
Fernandino, Maria
Filleter, Tobin
Firebaugh, Samara
Fischer, Andreas
Fischlechner, Martin
Fonollosa, J.
Fouillet, Yves
Fourati, Hassen
Fournel, F.
Frangi, Attilio
Frank, Hedrich
Galstian, Tigran
Garmire, David G.
Garstecki, Piotr
George, Sajan Daniel
Ghabcheloo, Reza
Ghassemali, Ehsan
Ghatkesar, Murali Krishna
Ghisi, Aldo
Ginn, James
Giorgio, Ivan
Giunta, Gaetano
Glynn, Macdara T.
Gomez, Frank A.
Goryachev, Maxim
Gossett, Daniel
Green, Nicolas G.
Greminger, Michael
Groche, Peter
Große, Sebastian
Guerrier, Stéphane
Guijt, Rosanne M.
Guldiken, Rasim
Gupta, Bhagwati
Guu, Y. H.
Han, Jae-Hung
Han, Lei
Hane, Kazuhiro
Hao, Guangbo
Hara, Motoaki
Harazim, Stefan M.
Hashiguchi, Gen
Hawkins, Aaron R.
He, Guohong
He, Liqun
He, Mei
Hemsel, Tobias
Henry, Manus
Herrera-May, A. L.
Higgins, Adam
Higgs, Gadryn
Hirai, Yoshikazu
Hisamoto, Hideaki
Hitt, Darren L.
Höflinger, Fabian
Hofmann, Ulrich
Hofmeister, William H.
Honeychurch, Kevin
Hornak, Larry A.
Hossack, John
Hossan, Mohammad
Hou, Ti-Kuang
Hsiao, Fu-Yuen
Hu, Zhongxu
Huang, Chih-Ching
Huang, Weimin
Huang, Yeou-Lih
Huang, Yue
Hughes, Michael P.
Hung, Kuo-Yung
Hung, Min-Sheng
Huo, Dehong
Hwang, Hyundoo
Hwang, Woo Suk
Ichikawa, Akihiko
Ikeda, Shoji
Ikehara, Tsuyoshi
Inokawa, Hiroshi
Iovenitti, Pio
Iqbal, Umar
Irimia, Daniel
Ishida, Tadashi
Ishikawa, Takuji
Italiani, Paola
Izquierdo, Ricardo
Jacobi, Ian
Jalabert, Laurent
Jang, Gunhee
Jang, Larry
Javanmard, Mehdi
Jensen, B. D.
Jeon, Tae-Joon
Jeong, Ok Chan
Jia, Yu
Jiang, Ruinian
Jiang, Xiaoning
Jiao, Lihong
Jiao, Zhenjun
Jiguet, Sebastien
Johnson, Tom
Jun, Martin
Kang, Chong Yun
Kang, Yuejun
Ke, Liaoliang
Keating, Adrian
Kelly, Jonathan
Kelly, Ryan
Khoshmanesh, Khashayar
Kietzig, Anne-Marie
Kim, Dae Eun
Kim, Dong Sung
Kim, Hoe Joon
Kim, Jungkyu
KIM, Kwang-Yong
Kim, Kyuman
Kim, Shin-Hyun
King, L. Brad
Kinzel, Edward C.
Kitsara, Maria
Ko, Seung Hwan
Kolb, Thorsten
Kooij, Srefan
Korvink, Jan G.
Kosa, Gabor
Koumoulos, Elias P.
Krull, Ulrich J.
Kumar, Aloke
Kwak, Moon K.
Kwong, Chan Pak
Lacarbonara, Walter
Lai, King Wai Chui
Lam, Raymond H. W.
Lee, Chang-Soo
Lee, Jeong Bong
Lee, Seok Jae
Lee, Woei Ming
Lee, Yung-Chun
Lei, Kin-Fong
Lévesque, Luc
Li, Feng-Ming
Li, Wen
Li, Yangmin
Li, Zheng
Liang, Jun-Rui
Liao, Yang
Lim, C. W.
Lima, Rui
Lin, Chih-Lang
Lin, Chih-Ming
Lin, Chih-Ting
Lin, Jr-Lung
Lin, Lih Y.
Lin, Long
Liu, Ai Qun
Liu, Bifeng
Liu, Chunhua
Liu, Haihu
Liu, Hong
Liu, Jing
Liu, K.
Liu, Shuo
Liu, Zhenyu
Longo, Domenico
Lou, Xia
Luo, Xiaolong
Luxey, Cyril
Ma, Hsi-Pin
Maasch, Matthias
Mace, Charles
Mackay, Ruth
Magda, Jules J.
Maharbiz, Michel
Mailly, F.
Malinauskas, Mangirdas
Malmstadt, Noah
Marcelli, Romolom
Marcos, Marcos
Marie, Rodolphe
Markx, Gerard H.
Maruyama, Takahiro
Masiero, Andrea
Mastrangeli, Massimo
Mathioulakis, Dimitris
Matiko, Joseph W.
Mauri, Roberto
Mcnamara, Shamus
Melvin, Adam
Miller, Phillip
Millet, Larry J.
Min, Sangkee
Minas, Graca
Minea, Alina Adriana
Misra, Sarthak
Mita, Agnès Tixier
Montes, Laurent
Moon, Chan-Woo
Moreno, Pablo
Morgan, Nicole Y.
Morshed, Bashir I.
Müller, Daniel J.
Mulvana, Helen
Mussetta, Marco
Myers, Richard
Nabki, Frederic
Nagl, Stefan
Nahar, Niru K.
Nakano, Kimihiko
Nanni, Emilio
Naseradinmousavi, P.
Nassiopoulou, Androula
Navin, Chelliah V.
Nemirovsky, Yael
Ng, Alphonsus
Nguyen, Duy-Son
Nirjon, Shahriar
Nisisako, Takasi
Niu, Xize
O’Mahony, Conor
Okino, Akitoshi
Oliveira, Mónica
Oliver, Nick
Olivier, Français
Ookawara, Shinichi
Orsini, Andrea
Oujja, Mohamed
Ozevin, Didem
Packirisamy, Muthukumaran
Paek, Jungwook
Palagi, Stefano
Pamplona, Vitor
Panchapakesan, Balaji
Panciroli, Riccardo
Pandraud, G.
Pane, Salvador
Pang, Wei
Pannasch, Sebastian
Papathanasiou, Athanasios
Parameswaran, Ash
Park, Jongho
Park, Kyung Hyun
Park, Soo-Young
Park, Sukho
Parker, Ted
Parthenios, John
Paust, Nils
Pawashe, Chytra
Peham, Christian
Pei, Ling
Pelosi, Paolo
Perez-Castillejos, Raquel
Perry, Sarah
Peyer, Kathrin
Pfeil, Shawn H.
Pierron, Olivier N.
Pisignano, Dario
Płaczek, Marek
Plagianakos, Fanis
Poenar, Daniel P.
Polimeno, Antonino
Pompa, Pier Paolo
Pope, Jodie G.
Potkay, Joseph
Previtali, Barbara
Priest, Craig
Propotny, Igor
Qiao, Lei
Qu, Hongwei
Raharijaona, Thibaut
Rakotondrabe, Micky
Raptis, Ioannis
Raskar, Ramesh
Ratcliff, Erin
Ravelet, Florent
Rebollar, Esther
Reed, Marc
Reichel, Erwin Konrad
Ren, Yong
Renaudin, Valerie
Retscher, Günther
Retterer, Scott D.
Rezai, Pouya
Rezk, Amgad
Riccobene, Giorgio
Richards, Cecilia
Rinke, Günter
Rivadeneyra, Almudena
Rocha, Luis
Roos, Wouter
Roper, Michael
Roth, Gunter
Ryckeboer, Eva M. P.
Saha, Sourabh K.
Sakar, Mahmut Selman
Sander, Dirk
Sangorrin, Victor Sans
Sankaran, Sindhuja
Schaefer, Hans-Eckhardt
Schertzer, Michael
Schlanbusch, Rune
Schomburg, Werner Karl
Schonbrun, Ethan
Schulze, V.
Scorsone, Emmanuel
Scotti, Gianmario
Seo, Tae-Seok
Shah, Lawrence
Shang, Xiaobang
Sharma, Mrigank
Shea, Herbert
Sheta, Bassem
Shih, Steve Chao-Chung
Shih, Wei-Chuan
Shih, Wen-Pin
Shikida, Mitsuhiro
Shimotsuma, Yasuhiko
Shin, Yong
Shui, Lingling
Shum, Ho Cheung
Smith, Lloyd M.
Sobot, Robert
Song, Young Min
Soria, Leonardo
Sosa González, Carlos J.
Spinello, Davide
Stavrakis, Stavros
Su, Chunyi
Suga, Tadatomo
Suh, Yong Kweon
Sukhorukov, Vladimir L.
Suleski, Thomas J.
Sun, Meng
Sun, Yuh-Chang
Sunmee, Park
Suzuki, Hiroaki
Suzuki, Kenichiro
Suzurikawa, Jun
Tabib-Azar, Massood
Takahashi, Yasuhiro
Takei, Atsushi
Takei, Kuniharu
Tamazin, Mohamed E.
Tan, Say Hwa
Tanaka, Yo
Tang, Fei
Tao, Li
Tay, Wee Beng
Teng, Tun-Chien
Tenje, Maria
Theodoly, Olivier
Thorpe, Stephen
Tian, Zengshan
Toonder, J. M. J. Den
Topuz, Fuat
Tóth, Eszter L.
Trabia, Mohamed
Triantafyllou, Michael S.
Tricoli, Antonio
Trigona, Carlo
Tsai, Chen S.
Tsai, Jeng-Han
Tsai, Scott S. H.
Tseng, Wei-Lung
Tsiourlis, George
Vellekoop, Michiel J.
Vettore, Antonio
Viktorov, Vladimir
Vladisavljević, Goran T.
Walczak, Rafał
Walton, Anthony J.
Wang, Chen-Hao
Wang, Michael
Wang, Ping
Wang, Wei
Wang, Yi
Wang, Zhenfeng
Wiegerink, Remco
Williams, Frances
Wu, Bian
Wu, Chih Yang
Wu, Jayne
Wu, Junru
Wu, Shin-Tson
Wu, Te-Ho
Wu, Zhengzheng
Wünsche, Michael
Xie, Huikai
Xiong, Xingguo
Xu, Chao-He
Xu, Jie
Xu, Qingsong
Xu, Zhang-Run
Xue, Wei
Yalisove, Steven M.
Yan, Jize
Yang, Bin
Yang, Charles Chun
Yang, Chii-Rong
Yang, Hyun-Ho
Yang, Lung-Jieh
Yang, Mengsu
Yang, Qiang
Yang, Ruey-Jen
Yang, Ruiguo
Yang, Xiaoxi
Yang, Ya-Ting
Yang, Yingchen
Yannis, George
Yao, Bo
Yasui, Takao
Yeo, Junyeob
Yin, Yuehong
Yossifon, Gilad
Yu, Chin-Ping
Yu, Xiao-Ying
Yuan, Junqi
Yuan, Xuebing
Zaghloul, M.
Zahn, Jeffrey D.
Zambelli, Tomaso
Zangle, Thomas
Zehetner, Christian
Zhang, Haixia(Alice)
Zhang, Ji
Zhang, Ketao
Zhang, Lan
Zhang, Shiyu
Zhang, Weiping
Zhang, Yong
Zhao, Boxin
Zhao, Xiangyong
Zhao, Xin
Zhao, Yi
Zhao, Yulong
Zhao, Zhan
Zheng, Rencheng
Zheng, Yuanjin
Zhou, Jian
Zhou, Qifa
Zhou, Yunshen
Zhu, Likun
Zhu, Yonggang
Zhu, Zhen
Zhu, Zhigang
Zhuang, Cheng-Xin
Zimmerman, Will
Zou, Min

